# Novel *Waddlia* Intracellular Bacterium in *Artibeus* *intermedius* Fruit Bats, Mexico

**DOI:** 10.3201/eid2112.150002

**Published:** 2015-12

**Authors:** Sebastián Aguilar Pierlé, Cirani Obregón Morales, Leonardo Perea Martínez, Nidia Aréchiga Ceballos, Juan José Pérez Rivero, Osvaldo López Díaz, Kelly A. Brayton, Alvaro Aguilar Setién

**Affiliations:** Washington State University, Pullman, Washington, USA (S.A. Pierlé, K.A. Brayton);; Unidad de Investigación Médica en Inmunología, Mexico City, Mexico (C. Obegón Morales, L. Perea Martínez, N. Aréchiga Ceballos, A. Aguilar Setién);; Universidad Autónoma Metropolitana Unidad Xochimilco, Mexico City (J.J. Pérez Rivero, O. López Días)

**Keywords:** Chlamydiales, Waddlia cocoyoc, Chlamydia-like organisms, bacteria, fruit bat, Artibeus intermedius, frugivorous, Mexico

## Abstract

An intracellular bacterium was isolated from fruit bats (*Artibeus intermedius*) in Cocoyoc, Mexico. The bacterium caused severe lesions in the lungs and spleens of bats and intracytoplasmic vacuoles in cell cultures. Sequence analyses showed it is related to *Waddlia* spp. (order Chlamydiales). We propose to call this bacterium *Waddlia cocoyoc*.

Because animals and humans have shared health risks from changing environments, it is logical to expand the perspective of public health beyond a single species. Bats are unique among mammals in their ability to fly and inhabit diverse ecologic niches. These characteristics together with their regularly large colonial populations highlight their potential as hosts of pathogens (*1*). Their role in disease epidemiology is supported by their susceptibility to different microorganisms such as bacteria, fungi, parasites, and viruses, as illustrated by the recent Ebola outbreak in West Africa (*2*). Previous and ongoing research is predominantly focused on viral agents, and the prevalence and effects of pathogenic bacteria in bats have been neglected (*3*).

*Artibeus intermedius* (the great fruit-eating bat) is a common frugivorous bat in the tropical Americas. Several pathogens of interest have been isolated from or detected in *Artibeus* spp. bats, including *Histoplasma capsulatum*, *Trypanosoma cruzi*, and eastern equine encephalitis, Mucambo, Jurona, Catu, Itaporanga, and Tacaiuma viruses (*4*–*6*), but their pathogenicity in bats is not known. In this study, a novel *Chlamydia*-like pathogenic bacterium was isolated from *A. intermedius* bats that were collected to characterize rabies virulence in a frugivorous bat species.

## The Study

Adult *A. intermedius* bats (n = 38) were captured in the municipality of Cocoyoc in the state of Morelos, Mexico, in May 2012. Animals were kept in captivity by following the Guidelines of the American Society of Mammalogists for the Use of Wild Mammals in Research (*7*). Bats were observed for 2 months to ensure that existing infectious diseases did not develop. No animals had rabies antibodies detectable through rapid fluorescent focus inhibition test. Animals were inoculated intramuscularly with rabies virus (vampire bat variant 5020, 1×10 ^5.34^Fluorescent Focus). After 5 days, an adult male exhibited emaciation, restlessness, and depression. On day 20, the animal could not fly and remained on the floor of the cage. Areas of pallor appeared on its wings (Technical Appendix Figure 1). The animal died on day 28. Testing showed negative results for rabies virus by direct immunofluorescence of brain tissue smears and by PCR of nervous tissue. Skin biopsies were taken from the wing lesions for histopathologic analyses and isolation.

**Figure 1:**
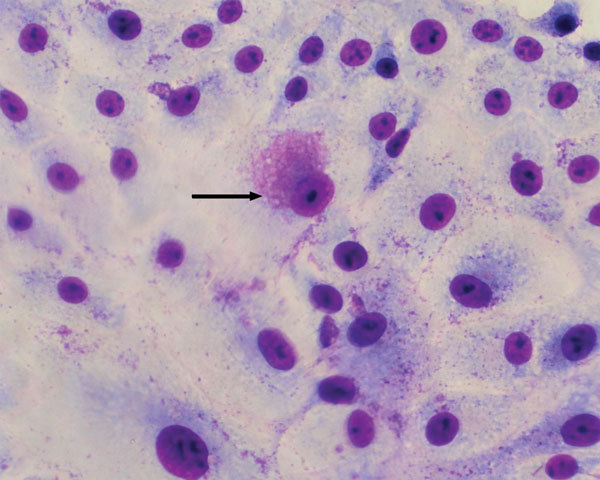
Bacterial cytoplasmic inclusions (arrow) in Vero cell cultures 72 h postinoculation with supernatant from homogenates of white spot lesion biopsies of adult *A*. *intermedius* bats in Mexico by using Diff–Quick stain (VWR International, Briare, France). Original magnification ×700.

Vero cells inoculated with supernatant from homogenates of white spot lesion biopsies showed cytopathic effect (CPE) within 72 to 96 h postinoculation. CPE consisted of lytic plaque formation. Acidophilic inclusions visible by using Diff-Quick (VWR International, Briare, France) staining were detected within 48 and 72 h postinoculation (Figure 1). Similar inclusions could be seen after inoculation of BHK21 cells. The microorganism could not be cultured on blood or chocolate agar, aerobically or anaerobically, when incubated for up to 7 days. 

Experimental inoculation was then established. Three bats that were seronegative for the isolated microorganism were inoculated intraperitoneally. The 3 animals were euthanized on days 5, 10, and 15. The bats euthanized on days 5 and 10 postinoculation showed signs of severe multifocal interstitial pneumonia (Technical Appendix Figure 2) and severe diffuse lymphoid hyperplasia in the spleen. On euthanization, the third bat showed signs of mild multifocal interstitial pneumonia and mild diffuse lymphoid hyperplasia in the spleen.

Two additional bats were inoculated subcutaneously; areas of pallor developed in the wing skin similar to those observed in the originally infected bat (Technical Appendix Figure 3). Mononuclear cells infected with bacteria were localized in skin (Technical Appendix Figure 4) and lung lesions of experimentally inoculated animals by immunofluorescence.

Histopathological findings in the areas of pallor through hematoxylin and eosin staining revealed the presence of mononuclear cell infiltrates in all subjects. Because of the resemblance of the wing lesions to those typically seen in white nose syndrome infection, which is caused by the fungus *Pseudogymnoascus destructans,* we applied periodic acid–Schiff staining to rule out fungal infection. No hyphae were identified.

Hyperimmune serum samples raised against the isolated bacteria neutralized the CPE of the bacteria up to a 1:719 dilution. Five (13%) of the 38 serum samples taken during captivity neutralized the CPE of the bacteria in dilutions ranging from 1:9 to 1:81. This result suggested circulation of this bacterium within the sampled population. All experimentally inoculated animals seroconverted. Serum samples from both animals that were inoculated subcutaneously neutralized the CPE of the bacteria up to a 1:27 dilution at day 28 postinoculation. Animals inoculated intraperitoneally and euthanized at 5, 10, and 15 days postinoculation neutralized CPE up to dilutions of 1:27, 1:27, and 1:81, respectively. No serum samples from the researchers who handled the bats showed seroneutralization activity.

**Figure 2:**
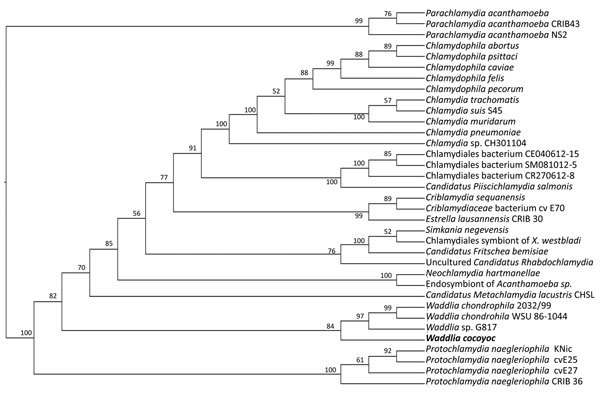
Phylogenetic relationships of bacterium newly identified in *Artibeus* *intermedius* fruit bats in Mexico (*Waddlia cocoyoc*, bold text), to other Chlamydiales. 16S sequences were used to infer relationships. *X. westbladi, Xenoturbella westbladi*.

DNA from skin biopsy samples and Vero and BHK 21 cells experimentally infected by using primers directed against domain I of the 23S gene of the family *Waddliaceae* yielded PCR products of the expected size (627 bp) (*8*). Vero cell culture was used to amplify the infection of the bacterial agent and DNA extracts were subjected to high throughput sequencing (SRA: PRJNA268154). Analysis of assembled contigs by using blastx (http://blast.ncbi.nlm.nih.gov/Blast.cgi) showed that sequences close to *Waddlia* spp. were abundant (43%) and were only surpassed by 2 sequences of primate origin (Technical Appendix Figure 5). A neighbor-joining phylogenetic tree with 10,000 replicates was built with 16S sequences from assorted members of the order Chlamydiales and the cultured microorganism. The Chlamydiales have evolved from a single genus to a diverse order including new families such as *Candidatus* Parichlamydiaceae and *Rhabdochlamiaceae* (*9*). Phylogenetic analyses revealed that the newly identified *Waddlia* sp. segregates with known *Waddlia* spp. Although the new *Waddlia* sp. fell in the same taxonomic unit, it is found in its own branch (Figure 2). This finding was confirmed by a maximum-likelihood phylogeny with approximate likelihood ratio test (Technical Appendix Figure 6).

## Conclusions

We report the isolation of a newly identified bacterial pathogen of *A. intermedius* bats and propose naming it *Waddlia cocoyoc*. The isolated bacterium was successfully grown in cell culture but not in inert bacterial growth media, suggesting dependence on host cells. Staining of inoculated cells revealed lysis and large intracytoplasmic vacuoles. Infected bats showed areas of pallor on the wings and had severe lesions in the lungs and the spleen. Histopathological analyses on the areas of pallor revealed mononuclear cell infiltrates in infected bats. Detection of the bacterium in lesion sites by immunofluorescence and PCR strongly suggests that it caused the observed pathogenesis. Phylogenetic analyses indicate that the pathogen is closely related to organisms in the family *Waddliaceae*.

Diversity in *Waddliaceae* increases as reports of new species surface. *Waddlia* spp. have been previously associated with Malaysian fruit bats (*10*). *W. chondrophila* has been isolated from aborted cattle fetuses in the United States (*11*), and was detected in a potoroo (*Potorous* spp.), a threatened marsupial native to Australia (*12*). Serologic evidence showed a substantive association between high titers of *W. chondrophila* antibodies and bovine abortion (*13*). In addition, *W. chondrophila* seroprevalence was found to be high in women who have had recurrent and sporadic miscarriages (*14*). *W. chondrophila* was also found in patients with community-acquired pneumonia (*15*). The host range and zoonotic potential of *Waddlia* spp. open multiple research avenues for this newly identified organism.

**Technical Appendix.** Untreated and treated lesions on wings of *Artibeus* *intermedius* fruit bats from Mexico infected with a bacterium related to *Waddlia* spp.; analysis of genetic sequences of the bacterium compared with other organisms; and phylogeny of the bacterial order Chlamydiales.

## References

[1] Kuzmin IV, Bozick B, Guagliardo SA, Kunkel R, Shak JR, Tong S, Bats, emerging infectious diseases, and the rabies paradigm revisited. Emerg Health Threats J. 2011;4:7159. http://dx.doi.org10.3402/ehtj.v4i0.7159 10.3402/ehtj.v4i0.7159 PMC3168224 24149032

[2] Leroy EM, Kumulungui B, Pourrut X, Rouquet P, Hassanin A, Yaba P, Fruit bats as reservoirs of Ebola virus. Nature. 2005;438:575–6. 10.1038/438575a 16319873

[3] Mühldorfer K. Bats and bacterial pathogens: a review. Zoonoses Public Health. 2013;60:93–103. 10.1111/j.1863-2378.2012.01536.x 22862791

[4] Calisher CH, Kinney RM, de Souza Lopes O, Trent DW, Monath TP, Francy DB. Identification of a new Venezuelan equine encephalitis virus from Brazil. Am J Trop Med Hyg. 1982;31:1260–72 . 7149112 10.4269/ajtmh.1982.31.1260

[5] McMurray DN, Thomas ME, Greer DL, Tolentino NL. Humoral and cell-mediated immunity to *Histoplasma capsulatum* during experimental infection in neotropical bats (*Artibeus lituratus*). Am J Trop Med Hyg. 1978;27:815–21 . 686249 10.4269/ajtmh.1978.27.815

[6] Reid JE, Jackson AC. Experimental rabies virus infection in *Artibeus jamaicensis* bats with CVS-24 variants. J Neurovirol. 2001;7:511–7. 10.1080/135502801753248097 11704883

[7] Gannon WL, Sikes RS. Guidelines of the American Society of Mammalogists for the use of wild mammals in research. J Mammal. 2007;88:809–23. 10.1644/06-MAMM-F-185R1.1 PMC5909806 29692469

[8] Everett KD, Bush RM, Andersen AA. Emended description of the order *Chlamydiales*, proposal of *Parachlamydiaceae* fam. nov. and *Simkaniaceae* fam. nov., each containing one monotypic genus, revised taxonomy of the family *Chlamydiaceae*, including a new genus and five new species, and standards for the identification of organisms. Int J Syst Bacteriol. 1999;49:415–40. 10.1099/00207713-49-2-415 10319462

[9] Stride MC, Polkinghorne A, Miller TL, Groff JM, Lapatra SE, Nowak BF. Molecular characterization of “*Candidatus Parilichlamydia carangidicola*,” a novel *Chlamydia*-like epitheliocystis agent in yellowtail kingfish, *Seriola lalandi* (Valenciennes), and the proposal of a new family, “*Candidatus* Parilichlamydiaceae” fam. nov. (order *Chlamydiales*). Appl Environ Microbiol. 2013;79:1590–7. 10.1128/AEM.02899-12 23275507 PMC3591964

[10] Chua PK, Corkill JE, Hooi PS, Cheng SC, Winstanley C, Hart CA. Isolation of *Waddlia malaysiensis*, a novel intracellular bacterium, from fruit bat (*Eonycteris spelaea*). Emerg Infect Dis. 2005;11:271–7. 10.3201/eid1102.040746 15752446 PMC3320453

[11] Rurangirwa FR, Dilbeck PM, Crawford TB, McGuire TC, McElwain TF. Analysis of the 16S rRNA gene of micro-organism WSU 86–1044 from an aborted bovine foetus reveals that it is a member of the order *Chlamydiales*: proposal of *Waddliaceae* fam. nov., *Waddlia chondrophila* gen. nov., sp. nov. Int J Syst Bacteriol. 1999;49:577–81. 10.1099/00207713-49-2-577 10319478

[12] Bodetti TJ, Viggers K, Warren K, Swan R, Conaghty S, Sims C, Wide range of *Chlamydiales* types detected in native Australian mammals. Vet Microbiol. 2003;96:177–87. 10.1016/S0378-1135(03)00211-6 14519335

[13] Dilbeck-Robertson P, McAllister MM, Bradway D, Evermann JF. Results of a new serologic test suggest an association of *Waddlia chondrophila* with bovine abortion. J Vet Diagn Invest. 2003;15:568–9. 10.1177/104063870301500609 14667020

[14] Baud D, Thomas V, Arafa A, Regan L, Greub G. *Waddlia chondrophila*, a potential agent of human fetal death. Emerg Infect Dis. 2007;13:1239–43. 10.3201/eid1308.070315 17953102 PMC2828094

[15] Haider S, Collingro A, Walochnik J, Wagner M, Horn M. *Chlamydia*-like bacteria in respiratory samples of community-acquired pneumonia patients. FEMS Microbiol Lett. 2008;281:198–202 . 10.1111/j.1574-6968.2008.01099.x 18312573

