# Supplementary material for: Novel Waddlia Intracellular Bacterium in Artibeus intermedius Fruit Bats, Mexico
Source: Emerg Infect Dis. 2015 Dec;21(12):2161–3. doi: 10.3201/eid2112.150002 (PMC4672442; doi:10.3201/eid2112.150002)
Supplement: Supplementary file 1 — Technical Appendix. Untreated and treated lesions on wings of Artibeus intermedius fruit bats from Mexico infected with a bacterium related to Waddlia spp.; analysis of genetic sequences of the bacterium compared with other organisms; and phylogeny of the bacterial order Chlamydiales. [file 15-0002-Techapp-s1.pdf]

Article DOI: http://dx.doi.org/10.3201/eid2112.150002

Novel *Waddlia cocoyoc* Intracellular Bacterium from Artibeus intermedius Fruit Bats, Mexico

Technical Appendix

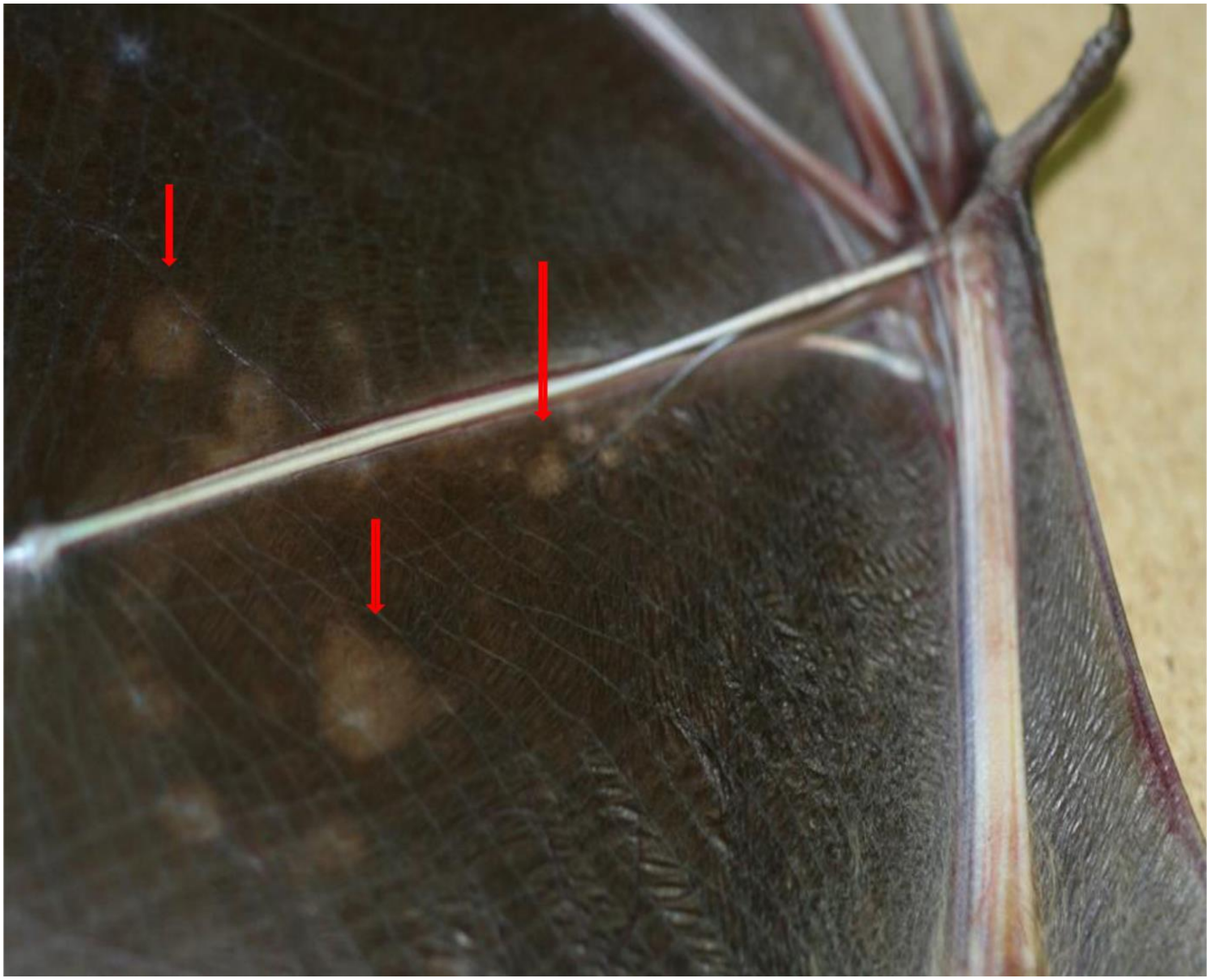

Technical Appendix Figure 1. White spot lesions on *Artibeus intermedius* bat wing skin (arrows).

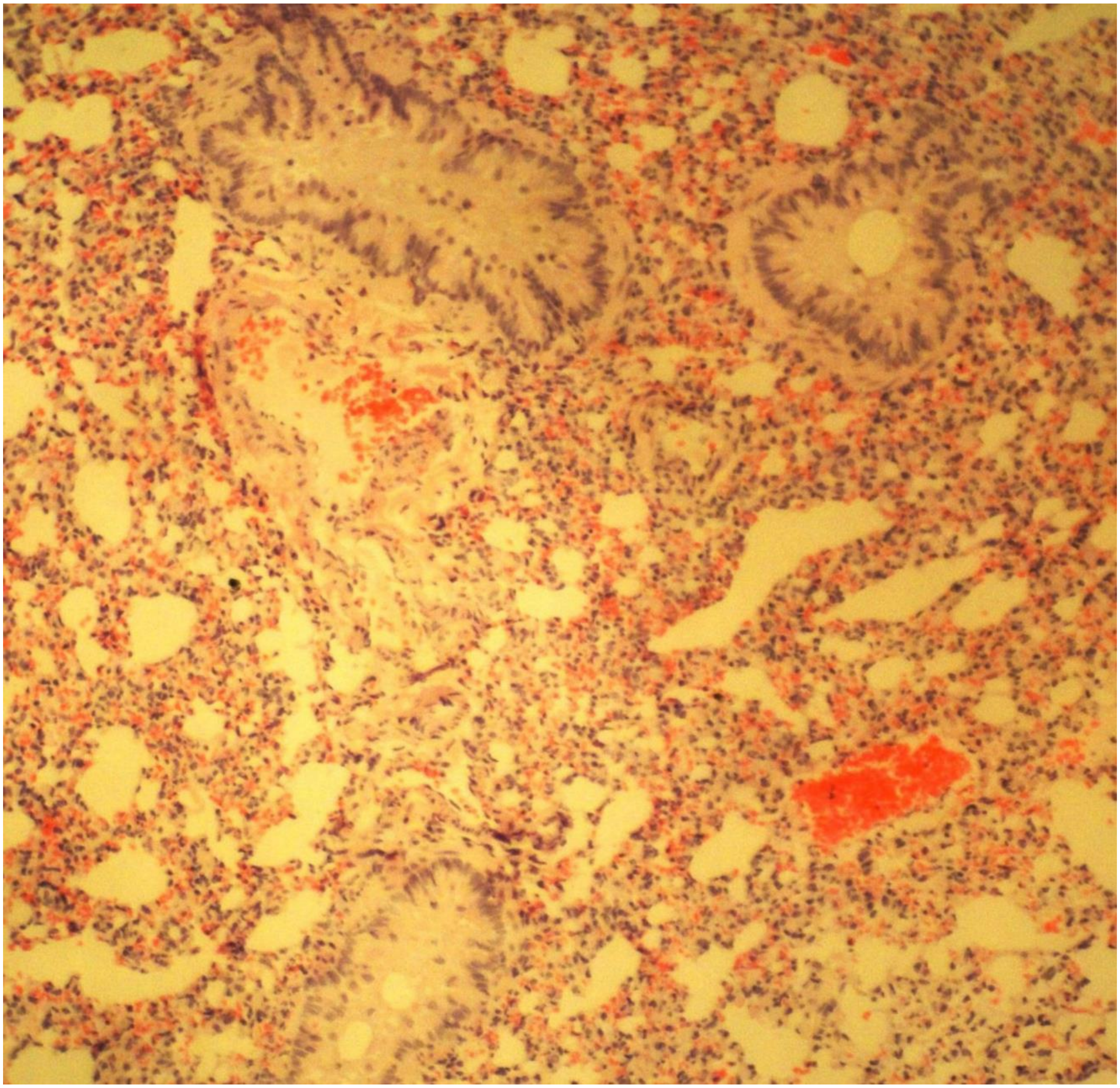

Technical Appendix Figure 2. Histological alterations (severe multifocal interstitial pneumonia) in an *Artibeus intermedius* bat, experimentally inoculated intraperitoneally with 10^3 50% tissue culture infectious dose 10 days postinoculation, stained by using hematoxylin and eosin, original magnification ×60.

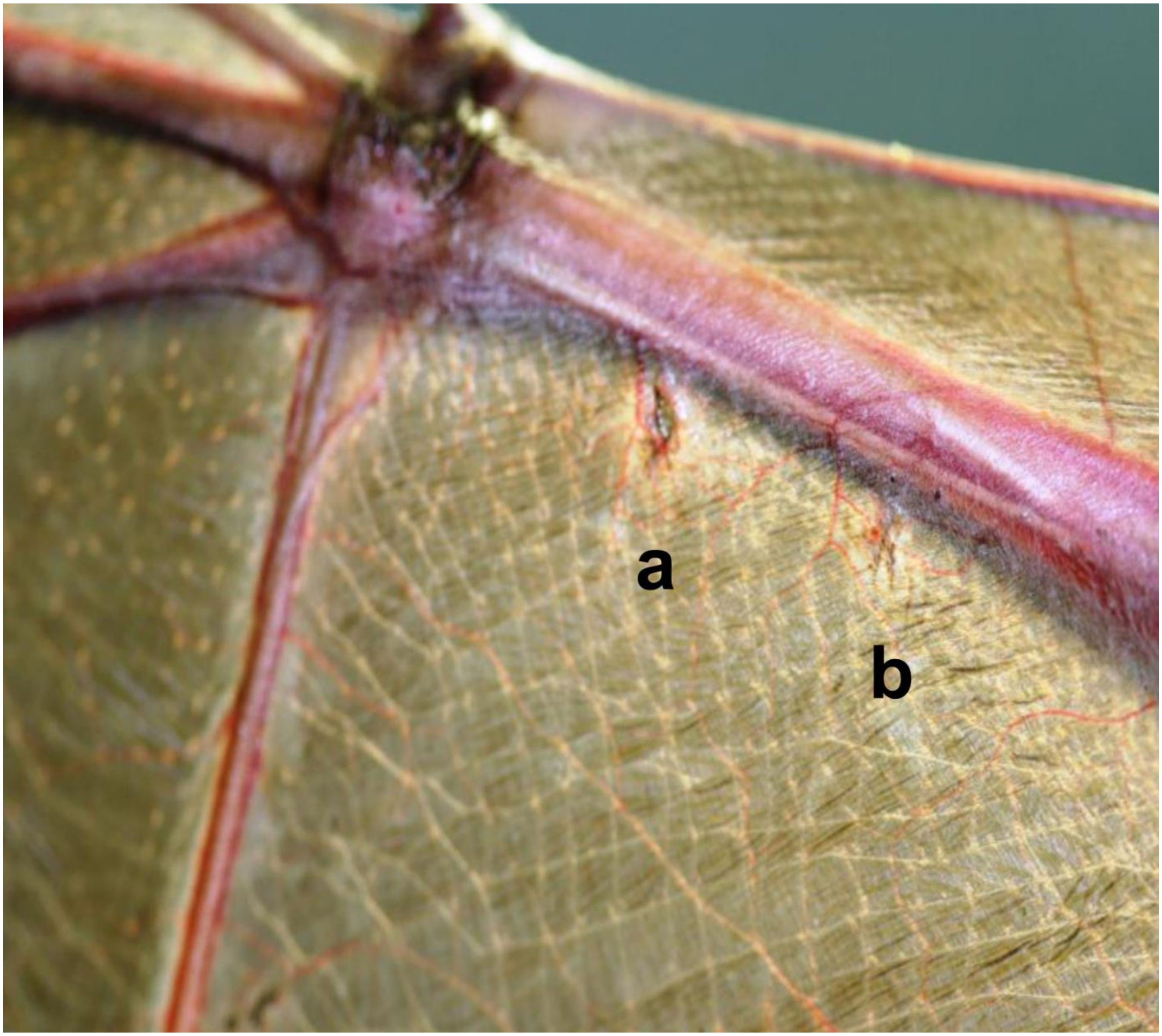

Technical Appendix Figure 3. White spot lesions in an *Artibeus intermedius* bat wing skin, 96 h after experimental inoculation with 10^5 50% tissue culture infectious dose of the (a) and 10^3 50% tissue culture infectious dose (b) of the newly identified bacterium.

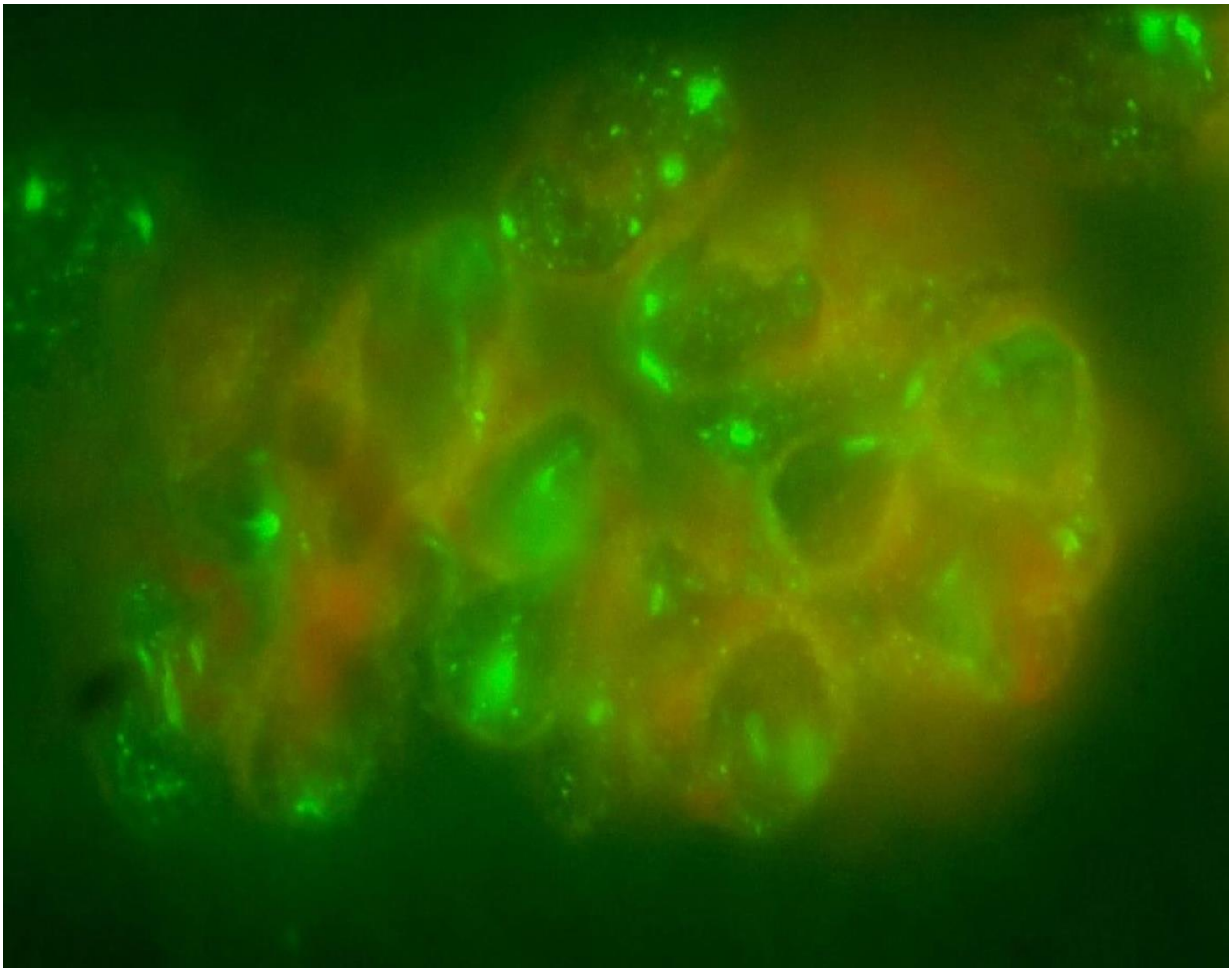

Technical Appendix Figure 4. Immunofluorescence of experimentally inoculated animals after using hyperimmune fserum against the newly isolated bacterium, original magnification ×1,000.

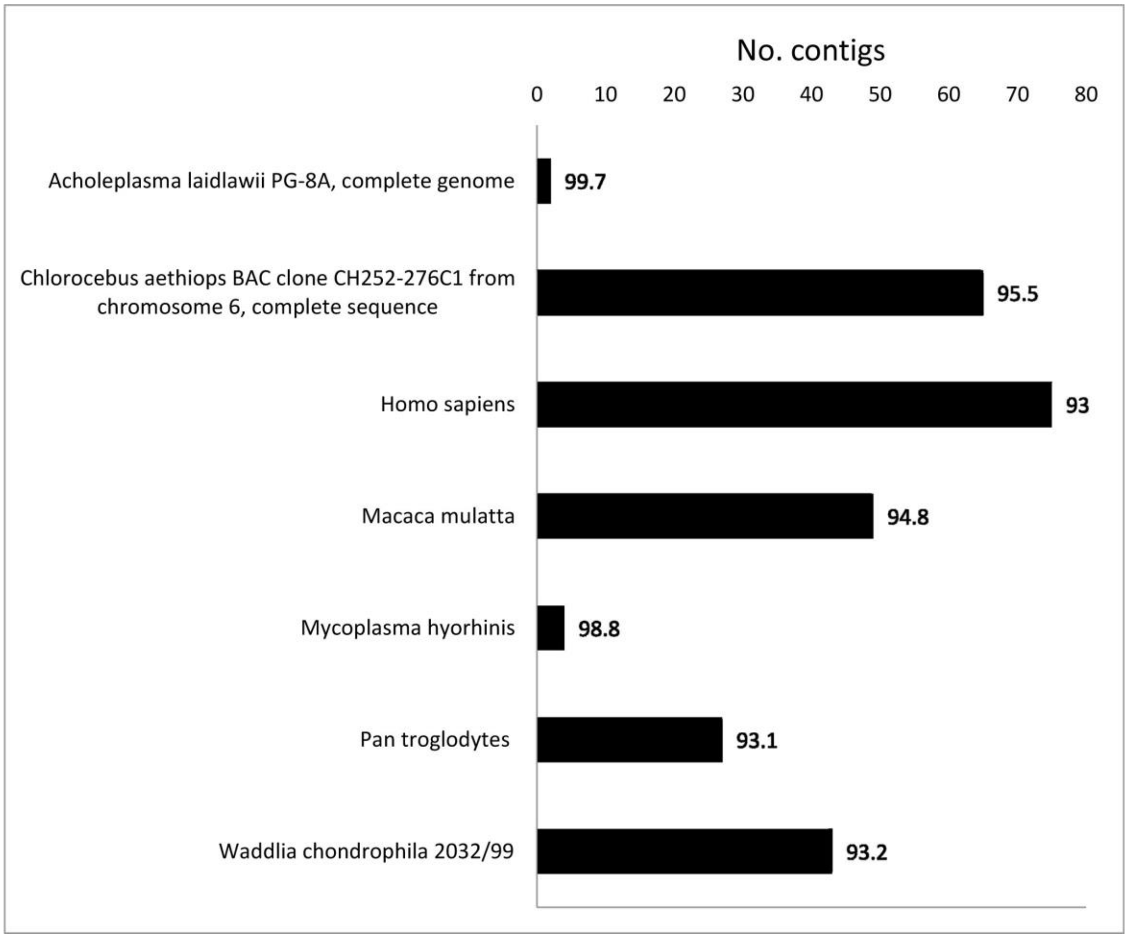

Technical Appendix Figure 5. The number of assembled contigs with high sequence identity to assorted organisms, determined by using the blastx algorithm (http://blast.ncbi.nlm.nih.gov/Blast.cgi) and shown per organism. Numbers in rows indicate the average % identity for the identified contigs.

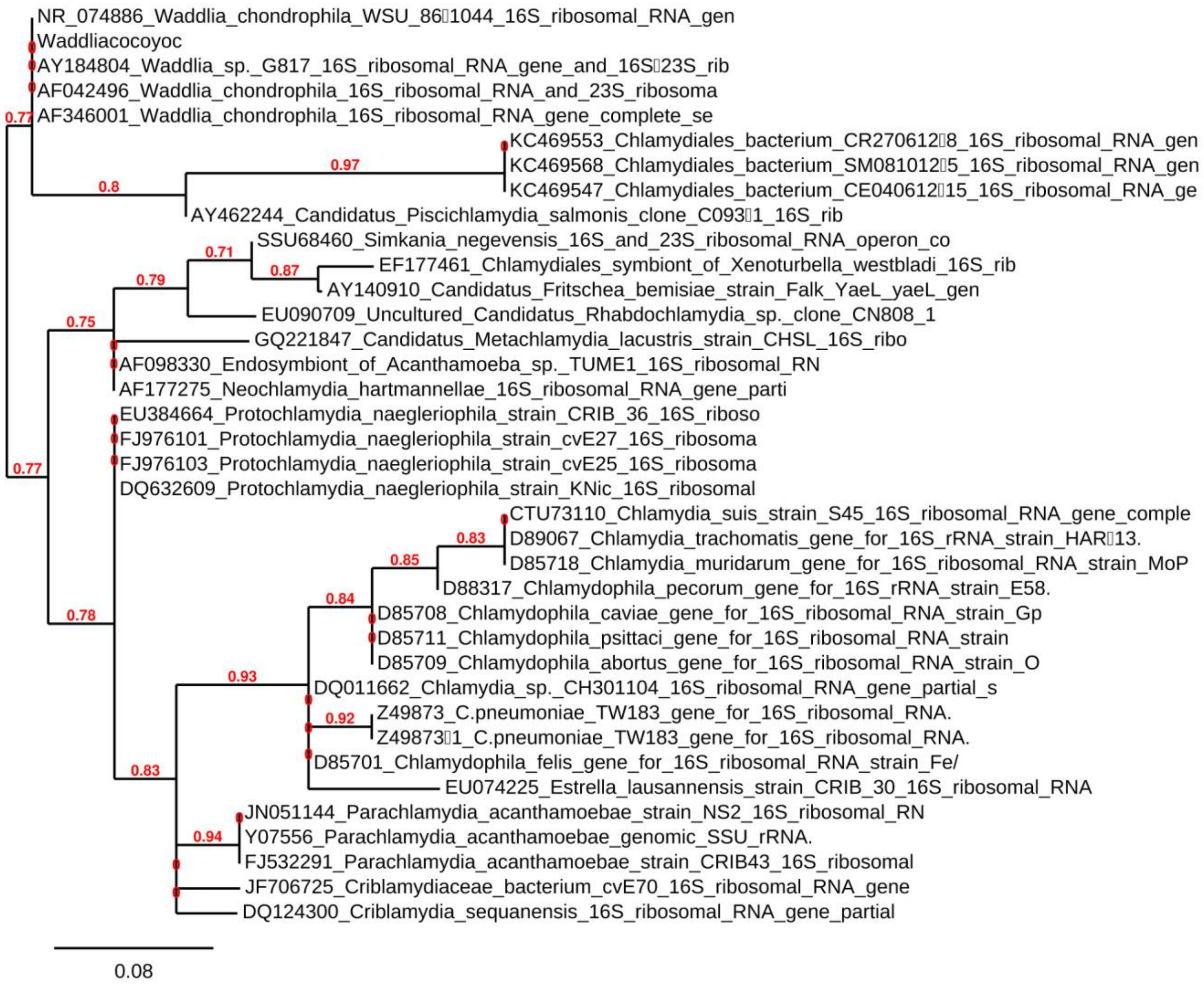

Technical Appendix Figure 6. Maximum-likelihood phylogeny with approximate likelihood ratio test for different members in the bacterial order Chlamydiales. Branch support values are shown in red. Species and GI numbers are shown. Scale bar indicates the scale of genetic change/expected substitutions per site.
